# Graph-Based Community Detection for Decoy Selection in Template-Free Protein Structure Prediction

**DOI:** 10.3390/molecules24050854

**Published:** 2019-02-28

**Authors:** Kazi Lutful Kabir, Liban Hassan, Zahra Rajabi, Nasrin Akhter, Amarda Shehu

**Affiliations:** 1 Department of Computer Science, George Mason University, Fairfax, VA 22030, USA; kkabir@gmu.edu (K.L.K.); lhassan1@gmu.edu (L.H.); nakhter3@gmu.edu (N.A.); 2 Department of Information Sciences and Technology, George Mason University, Fairfax, VA 22030, USA; zrajabi@gmu.edu; 3 Department of Bioengineering, George Mason University, Fairfax, VA 22030, USA; 4 School of Systems Biology, George Mason University, Fairfax, VA 22030, USA

**Keywords:** protein structure space, nearest-neighbor graph, community detection, decoy selection, template-free protein structure prediction

## Abstract

Significant efforts in wet and dry laboratories are devoted to resolving molecular structures. In particular, computational methods can now compute thousands of tertiary structures that populate the structure space of a protein molecule of interest. These advances are now allowing us to turn our attention to analysis methodologies that are able to organize the computed structures in order to highlight functionally relevant structural states. In this paper, we propose a methodology that leverages community detection methods, designed originally to detect communities in social networks, to organize computationally probed protein structure spaces. We report a principled comparison of such methods along several metrics on proteins of diverse folds and lengths. We present a rigorous evaluation in the context of decoy selection in template-free protein structure prediction. The results make the case that network-based community detection methods warrant further investigation to advance analysis of protein structure spaces for automated selection of functionally relevant structures.

## 1. Introduction

The (tertiary) structure which the peptide-bonded amino acids pack in three-dimensional (3d) space in a protein molecule is now recognized to be central to the biological activities of a protein in the living cell [1]. Due to this recognition, significant efforts in molecular biology are devoted to modeling a protein’s biologically active tertiary structure(s), also known as native structure(s). The plurality indicates that the native structure may often not be unique. The multiplicity of native structures may be harnessed by a protein to participate in several processes in the cell [2]. 

While great progress has been made in the wet-laboratory on protein (tertiary) structure determination (PSP), due to labor and cost demands, such efforts cannot keep up with the rapid advances in high-throughput sequencing technologies [3]. Computational methods offer a complementary approach. The most visible computational efforts are those under the umbrella of template-free PSP [4,5,6,7]. Provided a protein amino-acid sequence, these methods seek tertiary structures that are local minima of some selected energy/cost function, using this function as an indicator of biological activity. These functions are known to be inherently inaccurate; that is, structures that they report in the deepest local minimum they find may not be native, and known native structures may not reside in local minima of the employed function. Therefore, in addition to improving the accuracy of such functions, another central goal in template-free PSP is to generate/compute many structures, and then to employ additional criteria for discrimination of the native structure(s) among the ones computed. 

The contribution of the work presented in this paper is on the recognition goal, also known as the decoy selection problem; that is, given a computed set/ensemble of tertiary structures (to which one refers as decoys, as they hide among them the active structures), analyze this set to assist with recognition of the native structure(s). As our review of related work in Section 1.1 makes the case, decoy selection is a very challenging problem [8,9] for several reasons. One such reason is that software and hardware advances have resulted in an explosion of tertiary structure data. Template-free PSP methods can now generate dozens (and even hundreds) of thousands of tertiary protein structures for a given protein sequence in a matter of a few days, mainly by leveraging embarrassing parallelism in supercomputer architectures [3]. There is a growing need for methods that can uncover the organization of the protein structure space probed in silico by template-free PSP methods. Such organization can reveal, for instance, the grouping of structures in different thermodynamically stable states (corresponding to deep and broad basins/minima) and semi-stable states (corresponding to shallower basins).

In this paper, rather than designing criteria by which to analyze a computed tertiary structure, we propose techniques to elucidate the organization of the overall structure data provided by a template-free PSP method. For the purpose of evaluation, we focus here on tertiary, all-atom structure data obtained via the popular Rosetta *ab-initio* protocol, which represents a state-of-the-art and representative template-free PSP. Our approach is inspired by graph-based community detection of social and friendship networks. In a preliminary, proof-of-concept presentation of this approach in [10], we show how one can leverage a graph-based organization of tertiary structures to identify with community detection methods. In this paper, we extend and mature this work, and additionally incorporate the notion of energy in the construction of the network of computed structures. In addition to providing a principled evaluation of state-of-the-art, representative community detection methods for their ability to identify communities, we evaluate the utility of identifying communities for decoy selection. Among other results, we show that incorporation of energy in the construction of the network provides clear improvements for decoy selection.

The rest of this paper is organized as follows. Section 1.1 provides a summary of related work on decoy selection, followed by a detailed presentation of the results in Section 2 and a discussion in Section 3. The proposed methodology is described in Section 4.

### 1.1. Related Work

Research on decoy selection is regularly evaluated in the Critical Assessment of protein Structure Prediction (CASP) series of community-wide experiments [11]. Current decoy selection methods can be grouped into single-model, bag-of-models, quasi-single methods, and machine learning (ML) methods.

Single-model methods assess the quality of one tertiary structure at a time [12,13]. They do so by designing a physics- or knowledge-based energy/scoring function. Statistical functions are shown to be better able to distinguish native from non-natives structures [14]. In principle, all single-model methods employ a score threshold to determine which decoys pass the threshold and so can be deemed to be native-like. This approach is unreliable, as the threshold misses native structures or allows the inclusion of too many non-native ones [15].

In response, another approach is to ignore energy and instead cluster decoys by structural similarity [16,17]. The highest-populated clusters are offered as prediction (likely to contain native-like structures). Cluster-based decoy selection implements the bag-of-models approach that operates under the premise that decoys are randomly distributed around the “true answer,” which a consensus-seeking method ought to reveal. This may not be a valid assumption. Template-free methods employ heuristics that bias decoy generation away from uniform sampling of the structure space. Moreover, employed energy/scoring functions that are used to guide the sampling contain inherent biases that often invalidate entire regions of the structure space space. Cluster-based methods fail to pick up exceptionally good decoys and are challenged on hard targets where decoys can be very different from the known native structure(s), highly dissimilar, and sparsely sampled [11].

Quasi single-model methods combine strategies from single-model and bag-of-models methods, selecting first some high-quality structures to compare with the rest of the decoys [18]. These methods outperform single-model methods and consensus-seeking methods [19,20]. Recently, several machine learning (ML) models such as Support Vector Machines (SVM) [21], Neural Network [22], and Random Forest [23] have also shown to be competitive in decoy selection. Ensemble learning techniques outperform SVM learning [24] with or without statistical features. ML-based decoy selection represents a promising new thread of research in decoy selection.

## 2. Results

Our evaluation focuses on 10 target proteins of different folds and lengths (number of amino acids), listed in Table 1. The decoy ensemble of each target is generated from its amino-acid sequence via the Rosetta *ab-initio* protocol [4]. We make use of the Mason Argo supercomputing cluster to execute the protocol in an embarrassingly parallel fashion so as to obtain ensembles of at least 50,000 decoys per target. The actual ensemble sizes are shown in Column 5 in Table 1. The selected targets have known native structures to aid the evaluation. The Protein Data Bank [25] identifier (id) of the (crystallographic) native structure of each target is shown in Column 3 in Table 1.

The targets listed in Table 1 are divided into three categories (easy, medium, and hard) to indicate the quality of the Rosetta-generated decoy ensembles. This categorization emerges from analysis in terms of the lowest distance (measured via least root-mean-squared distance—lRMSD [26]) of all Rosetta-computed decoys from the corresponding native structure of a target. This distance is shown as min_dist in Column 6 in Table 1.

### 2.1. Evaluation Setup

For a given target protein (listed in Table 1), all decoys with lRMSD from the native conformation (in the corresponding PDB entry in Table 1) within a threshold dist_thresh are deemed *native*/near-native conformations. The threshold allows the population of a positive data set that is used for evaluation. As in previous related work [10], the threshold dist_thresh is set on a per-target basis, as there are targets on which Rosetta does not get close to 3 Å of the conformation in the target’s PDB entry: Specifically, if the lowest lRMSD (over all decoys) min_dist ≤0.7 (these are the easy cases in Table 1), dist_thresh is set to 2 Å. For medium-difficulty targets (0.7 Å< min_dist <2 Å), dist_thresh varies in the range 2−4.5 Å. We set the minimum dist_thresh to 6 Å if min_dist ≥2 Å (these are the hard cases). In [27], the interested reader can find the impact of different values of dist_thresh on the top cluster obtained via leader-clustering over these decoy datasets, which is used as guidance to determine dist_thresh values for construction of the nearest-neighbor graphs embedding decoy datasets.

The evaluation proceeds along two main dimensions.

First, we investigate the utility of organizing computed tertiary structures in nearest-neighbor graphs (nngraphs). While details can be found in Section 4, the main idea is that each computed structure becomes the vertex of the graph and is connected to other structures based on its lRMSD from other structures (employing either a maximum number of neighbors or a pre-determined distance threshold). In the directed version of the graph, edges have directionality, pointing from a vertex corresponding to a structure with higher energy (using Rosetta all-atom score score12) to a vertex corresponding to a structure with lower energy. Visually, these edges descend in the energy landscape probed by Rosetta via the computed decoys. On each setting, directed vs. undirected, we investigate state-of-the-art graph-based community detection methods and use benchmarks to evaluate these methods and select a few top-performing ones.

In a second dimension of our analysis, we focus on the top-performing community detection methods only and further evaluate the quality of the communities they detect in the context of decoy selection. Building on our prior work, we employ several selection criteria to select among the computed communities a few communities that are offered as prediction. These communities are evaluated according to several metrics we describe in detail in Section 4. We additionally propose new metrics, such as rank and entropy in this paper, and harden our observations via statistical significance tests. Additional evaluation is conducted to assess the impact of parameters in the performance of the presented approach, such as the impact of varying the number of nearest neighbors on the results.

### 2.2. Evaluation of Community Structure from Community Detection Methods

In our preliminary investigation in [10], we compare the performance of several state-of-the-art community detection methods along each of the benchmark metrics listed in Section 4 on undirected graphs constructed over the decoy datasets. The edge betweenness method is excluded from the comparison due to its ability to handle mainly sparse graphs. The evaluation in [10], which relates results on modularity, flake odf, conductance, and separability shows that the top performing method is Louvain, followed closely by Label Partitioning and Greedy Modularity Maximization (GMM), more information please see Supplementary Materials.

**Figure 1:**
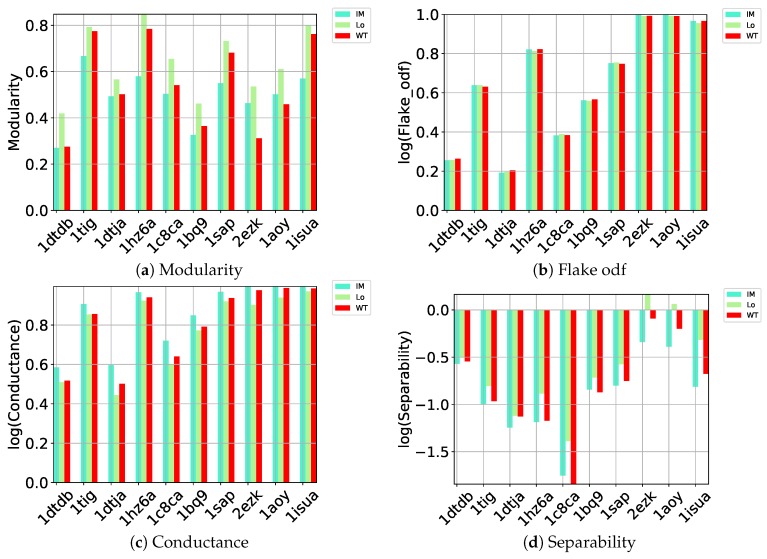
Comparison of community detection methods (encoded by different colors) on directed nngraphs embedding each of the 10 decoy datasets along (**a**) modularity, (**b**) flake odf, (**c**) conductance, and (**d**) separability.

Here we carry out a similar evaluation of these different community detection methods on the quality of the communities they detect (as gaged by the benchmark metrics listed in Section 4) on directed graphs constructed over the decoy datasets. Out of all community detection methods, only three implementations can handle directed graphs: Infomap, Louvain, and Walktrap. The performance of these three on (a) modularity, (b) flake odf, (c) conductance, and (d) separability is related in Figure 1.

Figure 1a relates the comparison along modularity and shows that Louvain achieves higher modularity than the other methods on each of the 10 test cases; as related in Section 4, higher modularity relates to better community structure. As described in Section 4, a better partitioning method achieves lower flake odf and lower conductance. Figure 1b–d draw the logarithm (base 10) of these metrics. Louvain, InfoMap, and Walktrap perform comparably according to flake odf, whereas, on conductance, Louvain achieves lower values than the other methods. Louvain outperforms the other methods on separability. As related in Section 4, the separability metric is higher in well-defined communities with many edges inside but fewer edges pointing to the rest of the network. This comparison again points to Louvain as the top community detection method to identify community structure in decoy data embedded in a directed nngraph.

### 2.3. Evaluation of Community Selection Strategies for Decoy Selection

Based on the results related above, we now focus the remainder of our evaluation on the communities obtained with the Louvain method on undirected and directed graph embeddings of decoy data, as well as on communities obtained with the GMM method (on undirected graph embeddings). Out of the communities into which a decoy dataset is partitioned by a specific community detection method, we select a top few via four different selection strategies. These are described in detail in Section 4. In summary, the selection strategies leverage the size of a community (the number of decoys in it) and its energy (defined as either the minimum or average energy over the decoys in it) to sort communities and then select the top *l* from the obtained sorted order, with *l* varying in {1,2,3}.

The selected communities are evaluated along the *n* and *p* metrics, also described in Section 4. In summary, these metrics evaluate the quality of a selected community in the specific context of decoy selection: *n* measures the percentage of near-native decoys in a selected set of decoys (the top community, or the combined decoys over the top l>1 communities) relative to the overall number of near-native decoys in the entire decoy dataset. Please note that a selected set of decoys can contain many near-native decoys but also many non-native ones. Therefore, *p* measures the percentage of near-native decoys in the selected set over the number of decoys in the set. This measure penalizes decoys with a lot of non-native decoys despite the number of near-native decoys. The idea is to relate the utility of a selected set of decoys that is offered as prediction. If *p* is high, then drawing at random from the selected set is likely to yield a near-native decoy, which can thus be offered as prediction. As related in further detail in Section 4, these metrics are inspired by machine learning performance metrics. 

We focus our evaluation on the top 1≤l≤3 (selected) communities, which we refer to as C1, C1−2, and C1−3 as *l* varies. The *n* and *p* metrics are calculated over the decoys in the combined set; that is, if we focus on the top three communities, the decoys in these three communities are combined, and the metrics of interest are computed over the resulting set. These metrics are related in Table 2, Table 3 and Table 4 for Louvain on undirected graphs, Louvain on directed graphs, and GMM on undirected graphs. In addition to *n* and *p*, these tables also report the size *s* of a selected set as a percentage over an overall decoy dataset. We note that for the selection strategies that use the energy of a community in addition to its size, Table 2, Table 3 and Table 4 only show results obtained when using the average energy over decoys in a community to associate an energy with a community. Obtained results are similar or worse when using the minimum energy.

**Figure 2:**
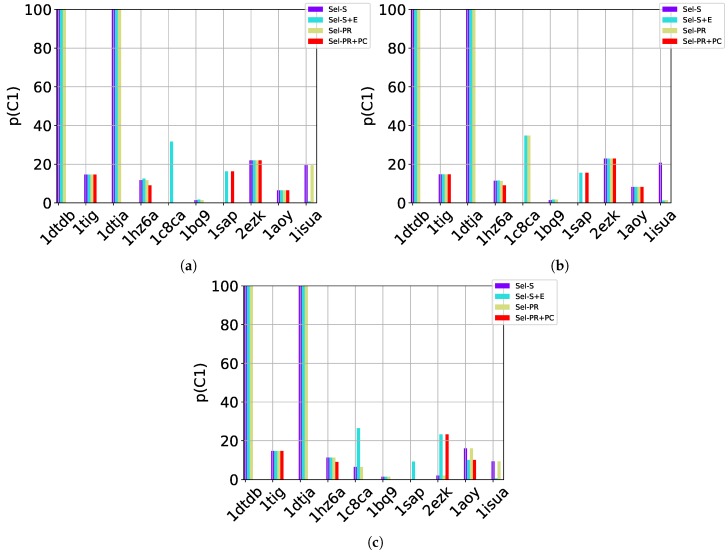
Comparison of the various selection strategies on the purity of the top community C1 selected over communities detected with the Louvain method on directed nngraph embeddings of decoy data in (**a**), the Louvain method on undirected nngraph embeddings of decoy data in (**b**), and the GMM method on undirected nngraph embeddings of decoy data in (**c**).

**Figure 3:**
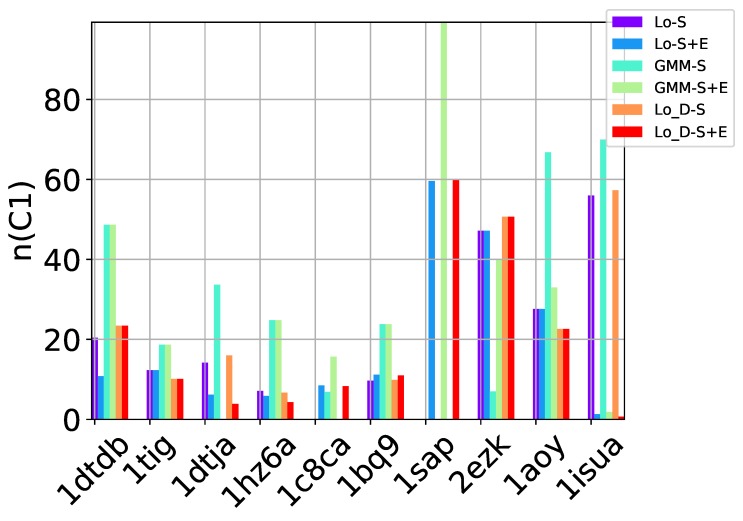

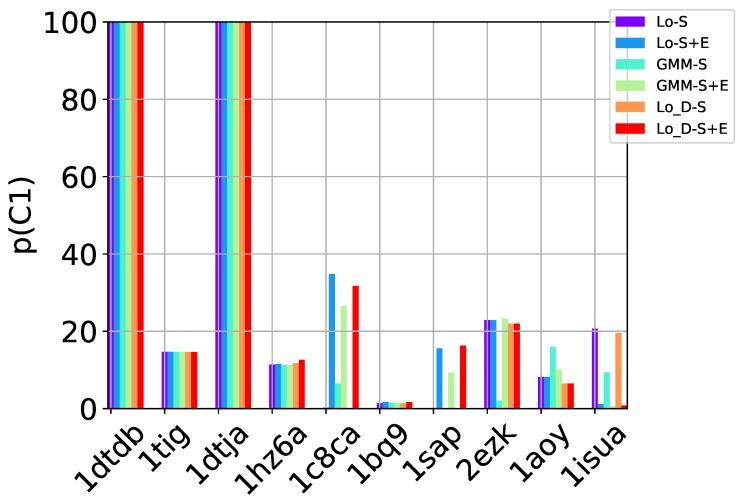
Comparison of community detection methods based on the quality of the top community selected by Sel-S and Sel-S+E. In the legend, Lo-D refers to the Louvain method applied to directed nngraphs that embed the decoy datasets. The -S and -S+E refer to the Sel-S and Sel-S+E selection strategies.

**Figure 4:**
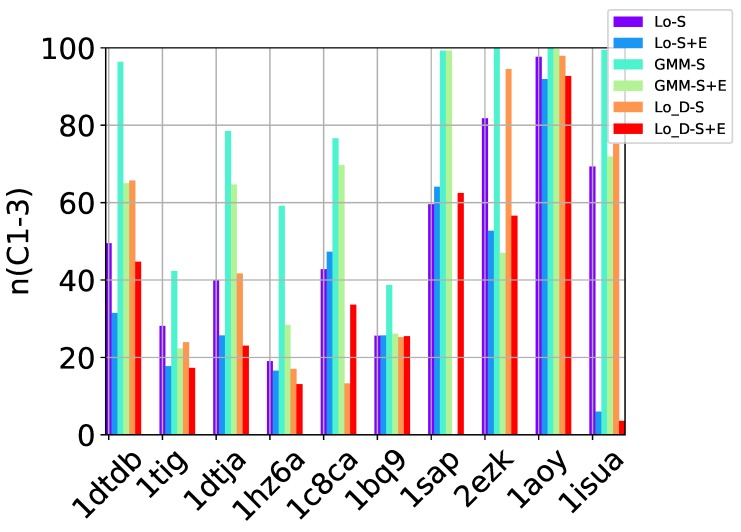

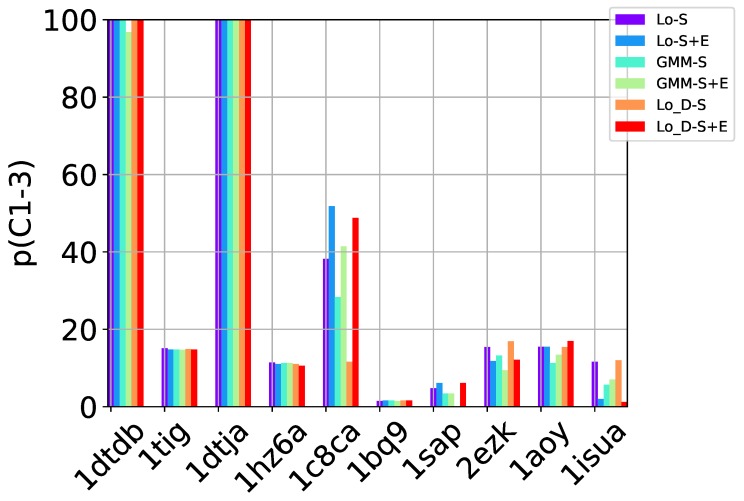
Comparison of community detection methods based on the quality of the top three communities selected by Sel-S and Sel-S+E. In the legend, Lo-D refers to the Louvain method applied to directed nngraphs that embed the decoy datasets. The -S and -S+E refer to the Sel-S and Sel-S+E selection strategies.

Table 2, Table 3 and Table 4 show that in many of the target proteins, the top communities have a high-percentage of near-native decoys. Purity is also high on the easy and medium targets, even reaching 100% purity on easy targets. The comparison of the four selection strategies to one another within each of the three community detection methods is made clearer in Figure 2a–c which show the purity of the top selected community, C1, by each of the four selection strategies over communities detected with the Louvain method on directed nngraph embeddings of decoy data in Figure 2a, the Louvain method on undirected nngraph embeddings of decoy data in Figure 2b, and the GMM method on undirected nngraph embeddings of decoy data in Figure 2c. This comparison shows that Sel-S+E and Sel-S outperform the other two selection strategies. Further comparison is provided in Figure 3, which shows *n* and *p* for C1 and in Figure 4, which does so for C1−3 selected by Sel-S and Sel-S+E over communities detected by any of the three community detection methods (Louvain over directed and undirected nngraphs and GMM over undirected nngraphs).

Taken altogether, these results suggests that a community-based, blind prediction method will offer as prediction only near-native decoys on ensembles of good-quality decoys (purity is as high as 100%). In particular, GMM seems well-suited to be used with either Sel-S and Sel-S+E. However, as the test cases become harder, Louvain yields higher purity, with the undirected and directed versions performing comparably. It is worth noting that prior work in [10] shows that community-based decoy selection performs better than (leader) clustering-based selection, particularly as the decoy datasets become more challenging.

#### 2.3.1. Rank-Based Comparison of Selection Strategies

We harden the above comparison of selection strategies via two sets of analyses.

First, we compare the four selection strategies based on the rank/position of the purest community in the sorted order they impose on detected communities. Table 5 reports the rank of the top-selected community (by each selection strategy) in a purity-based ordering (from high to low purity). The lowest rank over the selection strategies is highlighted in bold font. The results in Table 5 show that the lowest rank is obtained overall by Sel-S+E, which selects communities by size and energy (see Section 4 for a detailed description of the four selection strategies). Moreover, on the medium- and hard-difficulty datasets, the Louvain on directed and undirected and GMM behave comparably, with GMM outperforming the two other methods on the easy datasets.

Second, we conduct 1-sided and 2-sided statistical significance analysis via Fisher’s [28] and Barnard’s [29] exact tests on 2 × 2 contingency matrices. The analysis compares Sel-S+E to the other three selection strategies on the rank of the top-selected community in a purity-based ordering (from high to low purity). That is, 30 values are obtained for each selection strategy. Over each of the 10 decoy datasets, the rank of the top-community selected over those identified by Louvain on directed and undirected nngraphs and GMM on undirected nngraphs. Fisher’s exact test is conditional and widely adopted for statistical significance. Barnard’s test is unconditional and generally considered more powerful than Fisher’s test on 2 × 2 contingency matrices. We use 2-sided tests to determine which algorithms do not have similar performance and 1-sided tests to determine if Sel-S+E performs significantly better than the other selection strategies.

The top panel in Table 6 evaluates the null hypothesis that Sel-S+E does not provide the best rank, considering each of the other three selection strategies in turn. The bottom panel evaluates the null hypothesis that Sel-S+E does not provide a better rank with respect to another particular selection strategy, considering each of the other three in turn. The results in Table 6 show that the null hypothesis is rejected in both cases.

Table 7 shows a similar comparison for a 2-sided test. The results in Table 7 show that the null hypothesis is rejected in both cases. Taken together, the analysis confirms that Sel-S+E is the top performing selection strategy with regards to the rank of the the top-selected community in a purity-based order of detected communities.

#### 2.3.2. Impact of Graph Density on Purity

**Figure 5:**
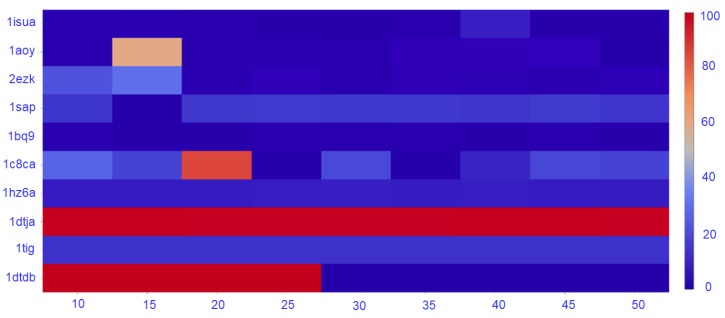
Purity for the range of k from 10 to 50.

The density of the nngraph embedding a decoy dataset impacts purity, as it ultimately impacts community detection. Therefore, here we conduct the following experiment. We vary the number of nearest-neighbors *k* that determines the number of edges that connect a vertex to other vertices. Specifically, *k* varies in {10,15,20,25,30,35,40,45,50}. In each case, a new nngraph is computed from a decoy dataset, and a community detection method is applied. Sel-S+E is applied over the detected communities, and the purity of the top community C1 is calculated. Figure 5 reports purity of the top community as a function of *k* only for the GMM, as the trend that is observed is similar for Louvain on directed and undirected nngraphs.

Figure 5 shows that the number of nearest neighbors has a great impact on the purity of the top community. As expected, the trend observed is that purity decreases as the number of nearest neighbors increase. For most of the datasets, the decrease starts after 20–25 neighbors, suggesting a value of k=20, which is the one we have used for the results reported in this section.

### 2.4. Entropy-Based Evaluation of Identified Communities

Finally, we pursue an orthogonal evaluation of the communities identified by the Louvain method on undirected and directed nngraph-embedded decoy datasets and GMM on undirected nngraphs. In particular, we investigate how the near-native decoys are spread among identified communities: are they mainly housed by a few communities, or are they spread over all identified communities? An entropy-based measure allows us to evaluate the heterogeneity of this distribution. Specifically, we define Entropy = $-\sum_{C}\left(n*\ln n\right)$ where *n* is the fraction of near-native decoys in a community ***C***. A low entropy value indicates that the near-native decoys are distributed among fewer communities, whereas a high entropy value indicates that the near-native decoys are spread over all or most of the communities. In the extreme, if all of the near-native decoys are present in a single community, then the value of entropy is 0 (the minimum possible value). A uniform distribution of near-native decoys over all communities yields the maximum value for entropy. 

Table 8 shows the entropies obtained by Louvain (on undirected and directed nngraphs) and GMM on each of the 10 test decoy datasets. The lowest value on each row is highlighted in bold font. The results show that the lowest entropy is obtained for GMM on the decoy dataset 1sap. Specifically, the second largest community among those identified by this method on this dataset contains 99.3% of the near-native decoys. Overall, GMM achieves the lowest entropy on each decoy dataset, as, due to its greedy nature, the method reports few, large communities that are likely to have many near-native decoys. Invariably, such communities have low purity, as related above. On the other hand, Louvain reports communities with higher purity, and the entropy values it obtains approach those of GMM as the datasets become harder. In particular, on the most difficult decoy datasets (the third category), Louvain on directed nngraphs reports lower entropy values than Louvain on undirected nngraphs.

## 3. Discussion

The findings show that community detection methods warrant further investigation for organizing protein structure spaces probed in silico and promise advancing decoy selection in template-free protein structure prediction. The evaluation related here makes the case that state-of-the-art community detection algorithms, such Louvain and Greedy Modularity Maximization, when coupled with simple selection strategies discriminating communities by size and energy, offer communities with a high purity for decoy selection. The presented results additionally suggest that on challenging decoy datasets, directed graph embeddings that further consider decoy energy may provide purer communities or better rank of the purest community.

The presented work motivates further algorithmic research in community detection algorithms for on-graph clustering of molecular structure data. The work opens additional lines of enquiry. For instance, the decoys in communities can be further assessed by different scoring functions for indicators of nativeness. The quality of the selected communities themselves can be further improved via a combination of unsupervised and supervised learning strategies.

The presented work focuses on an important sub-problem in template-free protein structure prediction. We note, however, that the work can also be applicable in other settings beyond template-free protein structure prediction. Many other structure modeling studies necessitate analysis of numerous structures generated in silico, whether of uncomplexed or complexed molecular systems. The work presented here may be useful in revealing the underlying organization of such data and capturing functionally relevant structural states.

## 4. Materials and Methods

We propose to leverage a graph-based representation of a computationally probed protein structure space as follows. A nearest-neighbor graph, which we define below, is used to embed computed structures of a protein molecule of interest. Utilizing ideas and techniques from network science and network community detection, the graph is then subjected to community detection methods. The latter group/organize structures into communities. In this paper, we first evaluate the obtained communities and then demonstrate how various ranking-based techniques that leverage properties of identified communities perform in automatically selecting communities more likely to contain functionally relevant structures in the context of decoy selection. We now describe the main steps of the proposed approach.

### 4.1. A Graph-Based Embedding of Decoys

We first note that the ideas laid out in this paper are general and extend beyond molecular structures. However, to directly connect with our objective of evaluating these ideas in the context of decoy selection, we refer to the data (the molecular structures) where we seek an underlying organization as decoys. 

As summarized above, a nearest-neighbor graph (nngraph) is employed to represent the structure space probed via computation. The graph encodes the proximity of decoys in this space. Let us denote the nngraph where the set of decoys are embedded as G=(V,E). The decoys populate the vertex set *V*. A local neighborhood structure is inferred for each decoy to populate the edge set *E*. This is based on proximity, measuring the distance between two decoys via root-mean-squared-deviation (RMSD). First, each decoy is superimposed over a decoy selected as reference (we arbitrarily choose as reference the first decoy). The superimposition minimizes differences due to rigid-body motions, as described in [26]. After this superimposition, the RMSD is then measured between every pair of decoys. Please note that superimposing all decoys to a reference decoy and then performing pairwise RMSD computations saves computational time. In contrast, seeking an optimal superimposition for each pair of decoys would result in quadratic (rather than linear) running time. Once such distances are available for every pair of decoys, the neighbors of each vertex u∈V are other vertices v∈V such that d(u,v)≤ϵ, where ϵ is a user-defined parameter that controls the radius of the neighborhood. A vertex is connected via an edge to each of its neighbors determined in this manner. We note that proximity query data structures (such as kd-trees and others) allow efficiently extracting the nearest neighbors of a vertex.

It is worth noting that the value of ϵ is an important consideration. A small value may result in a disconnected graph. This can be remedied by initializing ϵ to some initial value $\epsilon_{0}$ and then increasing it by δϵ over a maximum number $n_{\epsilon }$ iterations, while at the same time controlling the density of the nngraph via a parameter *k*. This parameter specifies the maximum number of neighbors allowed per vertex. In this way, only vertices with no more than *k* neighbors gain neighbors after each iterative increment of ϵ. Please note that k allows controlling the density of the graph. 

We note that alternative constructions of nngraphs use *k* directly rather than ϵ. Indeed, Section 2 presents an evaluation in terms of *k*. However, in molecular structure data that may be the result of non-uniform sampling, specifying *k* may result in connecting structures that are very different from each-other (that is, not in proximity).

We note that in what is described above, the nngraph is undirected. Directionality can be easily added to additionally include the role of energy in the embedding of a decoy dataset in a discrete structure, such as a graph. An edge can be directed from a vertex corresponding to a decoy with higher energy to a neighboring vertex corresponding to a decoy with lower energy.

Whether undirected or directed, the nngraph can now be investigated for its organization via community detection methods. We consider 7 representative, state-the-art methods, described below.

### 4.2. Community Detection Methods

**Edge betweenness (Girvan-Newman):** This approach was introduced to sidestep the drawbacks of hierarchical clustering. It operates based on the intuition that edges linking the communities are anticipated to possess high edge betweenness, which generalizes Freeman’s betweenness centrality [30] from vertices to edges. To reveal the underlying community structure of the network, the Girvan-Newman method successively removes edges with high edge betweenness. Measuring edge betweenness takes O(|E|·|V|) time. Since this step has to be carried out repeatedly (for each edge), the entire approach runs in $O(|E{|}^{2}\cdot |V)$ time.

**Leading Eigenvector (LE):** The prime objective of this method is modularity maximization (in terms of the eigenspectrum of modularity matrix) across possible subdivisions of a network [31]. With repeated divisions, the method discovers a leading eigenvector that partitions the graph into two subgroups; the goal of maximal improvement of modularity is achieved at every step. This process terminates when modification of modularity in the sub-network starts being negative. In fact, the method is associated with additional outcomes: a spectral measure of bipartite architecture in the network and a centrality measure to detect the vertices holding nuclear positions in communities. In general, the partitioning step takes O(|V|(|E|+|V|)) time.

**Walktrap (WT):** This method employs random walks to take into account the architectural resemblance between vertices (or groups of vertices). The underlying intuition is that vertices that are within the same community are supposed to have shorter distance for random walks [32]. The methods administers an agglomerative approach which starts from |V| communities (reduced to singleton clusters) and hierarchically merges two adjacent communities at each step. This is an effective approach to handle dense subgraphs of sparse graphs, which is most often the case for real-world complex networks. The method runs in time $O(|E||V{|}^{2})$ and space $O(|V{|}^{2})$ in the worst case.

**Label Propagation (LP):** This method is based on the intuition that each vertex in the network is supposed to follow the majority of its neighbors while joining a community [33]. The method aims robust use of the network infrastructure instead of a predefined objective function (to optimize) or *a-priori* information on the communities. At the beginning, a unique label is assigned to each vertex; that is, the method initializes |V| singleton communities. In progressive steps, adoption of a label comes into play for each vertex depending on the label possessed by the majority of its neighbors at that instant. This iterative process effectively performs the task of label propagation through the network and helps to form a consensus on a unique label for densely connected vertices. The process halts when each vertex and most its neighbors have an identical label. The algorithm takes linear time in the number of edges (O(|E|)). 

**Louvain (Lo):** This is a heuristic-based method focusing on modularity optimization [34]. The method consists of iterative repetition of two stages. The first stage deals with the initial partition, where each vertex is assigned to a unique community (singleton communities). Modularity gain is measured by assigning a vertex to a neighbor community so as to exclusively search for the way to maximize positive gain. The order in which vertices are explored does not affect modularity but may increase computation time. The second stage commences with the construction of a new weighted network, whose vertices are the communities generated by the first phase. The above process continues until maximum modularity is achieved.

**InfoMap (IM):** This method identifies communities by using random walks along with information flow analysis [35]. The vertices and their connections are decomposed into modules to represent the network in such a way that maximizes the amount of information in the actual network. The method tries to assign codewords to vertices; the process is efficient in terms of the dynamics on the network. A signal is transmitted to a decoder (via a limited capacity channel) who tries to decode the message, as well as to form viable candidates for the actual network. The lower the number of candidates, the more information about the actual network has been transmitted. The method runs in O(|E|) time.

**Greedy Modularity Maximization (GMM):** This is a hierarchical agglomeration method that makes use of a greedy optimization approach. The underlying assumption is that high values of modularity are associated with good communities [36]. Initially, each vertex itself forms a community. Then, vertices of two communities are combined together in a way that yields maximum modularity gain. This step is repeated (|V|−1) times. The process is represented as a hierarchical tree-like structure (a dendrogram), whose end-nodes represent the vertices of the actual network, and the internal vertices correspond to the connections; that is, the dendrogram shows a hierarchical decomposition (level-wise) of the network into communities. The method runs in O(|E|dlog|V|) time, where *d* is the depth of the dendrogram representing the network’s community architecture.

### 4.3. Metrics for Evaluating Community Detection Methods

A comprehensive list consisting of 15 community-recommended metrics has been considered to assess the community detection methods summarized above [37]. We note that the following metrics are scoring functions that perform mathematical formalization of the community-wise connectivity structure of a provided set of vertices and identify communities as high-scored sets. To summarize these metrics, let us consider a graph G(V,E) with n=|V| vertices and m=|E| edges, and a community is defined as a set *S* of $n_{S}$ vertices and $m_{S}$ edges. 

**Fraction Over Median Degree (fomd):** Let the degree of *u* for each vertex u∈S be denoted by d(u), and let $d_{m}$ be the median across the degrees d(u). Then, fomd is determined as the fraction of vertices in *S* with an internal degree greater than $d_{m}$; that is, $f\left(S\right)=\frac{|\{u:u\in S,|\left\{\left(u,v\right):v\in S\right\}|>d_{m}\}|}{n_{S}}$. The denser and more cohesive the communities, the higher the associated fomd scores.

**Max odf (out degree fraction):** Max odf evaluates the maximum ratio of edges of a vertex in community *S* which point outward from *S*. That is, $f\left(S\right)=\max_{u\in S}\frac{|\{(u,v)\in E:v\notin S\}|}{d(u)}$. According to Max odf, a community is characterized as a set of vertices that connect to more vertices within the set than to vertices outside of it. As a result, better communities are associated with lower Max odf scores.

**Triangle(Triad) Participation Ratio (tpr):** Let $T_{c}$ denotes the number of vertices which form a triangle in *S*. The tpr metric measures the ratio of vertices belonging to a triangle and can be formulated as: $f\left(S\right)=\frac{|\{u:u\in S,\{(v,w):v,w\in S,(u,v)\in E,(u,w)\in E,(v,w)\in E\}\neq \emptyset \}|}{n_{S}}$. Better community clustering yields higher tpr scores. 

**Internal Edge Density:** For a set *S*, let us denote the maximum number of possible edges by $m_{Smax}=n_{S}\left(n_{S}-1\right)/2$. The internal edge density is the ratio of the edges that are actually in *S*, denoted by $m_{S}$, over $m_{Smax}$; that is, $f\left(S\right)=\frac{m_{S}}{n_{S}\left(n_{S}-1\right)/2}$. This metric represents the internal connectivity of a cluster (community) and a higher score indicates that there are more connections within the vertices of that community. 

**Average Internal Degree:** This metric determines the average internal degree of the members of set *S* and can be formulated as: $f\left(S\right)=\frac{2m_{S}}{n_{S}}$. The denser a community, the higher its average internal degree score. 

**Cut Ratio:** Let $C_{S}$ denotes the edges that are going outward from a set *S*. The cut ratio score measures the ratio of $C_{S}$ over all possible edges and is defined as: $f\left(S\right)=\frac{C_{S}}{n_{S}\left(n-n_{S}\right)}$. Better communities are associated with lower scores. 

**Expansion:** This metric calculates the number of edges (for each vertex) going out of a set *S* and can be formulated as: $f\left(S\right)=\frac{C_{S}}{n_{S}}$. Lower scores correspond to better communities. 

**Edges Inside:** This metric measures the internal connectivity of a set *S* as $f\left(S\right)=m_{S}$. Better communities are related with higher scores.

**Conductance:** This metric is based on the combination of internal and external connectivity and is measured as: $f\left(S\right)=\frac{C_{S}}{\left(2m_{S}+C_{S}\right)}$. Lower scores relate with well-separated communities. 

**Normalized Cut:** This metric is defined as: $f\left(S\right)=\frac{C_{S}}{\left(2m_{S}+C_{S}\right)}+\frac{C_{S}}{2\left(m-m_{S}\right)+C_{S}}$. The metric has the special property that concurrently meets the two following objectives: maximization of dissimilarity across communities and minimization of overall similarity (eschewing the unnatural bias for breaking up small sets). Lower values of normalized cut maintain balance between these two objectives.

**Coverage:** This metric measures the ratio of the number of intra-community edges to the number of edges in the graph and is defined as: $f\left(S\right)=\frac{\omega (C)}{\omega (G)}$. Here, $\omega \left(C\right)=\sum_{i=1}^{k}\omega \left(E\left(v_{x},v_{y}\right)\right);v_{x},v_{y}\in C_{i}$. Higher coverage values indicate that there are more connections within communities rather than edges linking various communities. In fact, the ideal scenario is that communities are completely separated from one another, which would correspond to a coverage of 1 (the maximum possible value). 

**Average odf** This metric provides the average ratio of edges that point outward of *S* over vertices in *S* and is defined as: $f\left(S\right)=\frac{1}{n_{S}}\sum_{u\in S}^{}\frac{|\{(u,v)\in E:v\notin S\}|}{d(u)}$. Lower values of average odf relate with better communities. 

**Modularity:** This metric is based on the network model and determines the difference between the number of edges within *S* and the anticipated number of such edges in a random graph of exactly the same degree sequence. Modularity can be defined as: $f\left(S\right)=\frac{1}{4}\left(m_{S}-E\left(m_{S}\right)\right)$. Higher values of modularity correspond to denser connections within a community than anticipated at random. 

**Flake odf:** This metric combines internal and external connectivity and determines the fraction of the number of vertices with fewer connections within the community than with the outside. Flake odf is defined as: $f\left(S\right)=\frac{|\{u:u\in S,|\{(u,v)\in E:v\in S\}|<d(u)/2\}|}{n_{S}}$. Better communities are associated with higher values.

**Separability:** This is a community-goodness metric [37] based on the intuition that good communities are well-separated (have relatively few edges from set *S* to the rest of the network). Separability finds the ratio between edges pointing in and outside of the set *S* and is defined as: $f\left(S\right)=\frac{m_{S}}{C_{S}}$. Higher value indicate better communities. 

We note that these metrics can be grouped into four major classes [37]: metrics based on internal connectivity (fraction over median degree, triangle participation ratio, internal edge density, average internal degree, edges inside), metrics based on external connectivity (cut ratio, expansion), metrics based on internal-external connectivity (conductance, normalized cut, max odf, average odf, flake odf), and metrics based on the network model (modularity).

### 4.4. Community Selection for Decoy Selection

The methods described above organize decoys into communities (more generally, groupings) by using ideas from network community detection. In previous work [27], we have used ideas from statistics and computational geometry to group molecular structures into energy basins, leveraging the concept of the energy landscape. In that work, ranking-based methods are also debuted that evaluate groupings based on group-level characteristics and use these characteristics to rank groupings. Rankings provide a straightforward approach for decoy selection, and the quality of top-rank, selected groupings can be evaluated in the specific context of decoy selection.

Here, building on work in [27], we summarize what group-level characteristics can be associated with communities. They fall into three categories: size, energy, and hybrid characteristics. The size of a community is the number of decoys/vertices in it. Energy can also be associated with a community. We note that the decoys we consider here are generated from template-free methods, which pursue an optimization approach that seeks to minimize the interatomic energy in a structure via a selected energy function. The result is that each decoy has an associated energy value. Given the energies of decoys in a community, the energy of a community can be defined as the minimum energy over all decoys in it or the average over the energies of the decoys in it.

Whether size or energy, a selection technique ranks (via sorting) the communities and selects the *c* top-rank communities, offering them as “prediction” for where native and near-native structures reside. We refer to the size-based ranking/selection strategy as Sel-S. We recall that considering only energy would promote a significant number of false positives, as it is well known that protein energy functions are inherently inaccurate (which is the reason decoy selection remains challenging, as summarized in Section 1). Therefore, we consider both size and energy together in a second selection strategy to which we refer as Sel-S+E; we consider the l>c largest communities and then re-sort them from lowest to highest energy, selecting the top *c* of them for prediction.

Hybrid characteristics consider both size and energy but additionally take into account the possibility that size and energy are possibly conflicting optimization criteria. Since solutions minimizing all conflicting objectives simultaneously are typically non-existent, Pareto-optimal solutions are sought. A Pareto-optimal solution cannot be improved in one objective without sacrificing the quality of at least one other objective. That is, a solution $S_{1}$ *Pareto-dominates* another solution $S_{2}$ if the following two conditions are satisfied: (1) For all optimization objectives *i*, $score_{i}\left(S_{1}\right)\geq score_{i}\left(S_{2}\right)$; (2) For at least one optimization objective *i*, $score_{i}\left(S_{1}\right)>score_{i}\left(S_{2}\right)$. 

Based on the above, two additional quantities, Pareto Rank (PR) and Pareto Count (PC), can be associated with each community *C*. These two quantities employ the concept of dominance, summarized above. PR(*C*) is the number of communities that dominate *C*. PC(*C*) is the number of communities that *C* dominates. It is now straightforward to use these two new, hybrid characteristics, in the same ranking-based manner. We just note that in Sel-PR, the communities are sorted by low to high PR values; in Sel-PR+PC, PC is additionally considered as follows: Communities with the same PR value are additionally sorted from high to low PCs.

### 4.5. Evaluating Selected Communities

We note that the ranking-based techniques described above complete an unsupervised learning framework, where we first organize decoys into communities and then automatically select a set *S* of decoys from the top *c* selected communities to offer as “prediction”. As we relate in Section 2, *c* can be varied so as to evaluate not only the top community but to additionally extend the analysis to the top c>1 communities.

The set *S* of decoys offered as prediction can now be evaluated in the presence of a known native structure. The native structure is treated as the ground truth. Decoys within a dist_thresh RMSD of the native structure are considered near-native and are labeled as true positives (TP). We delay details on the selection of dist_thresh and its impact on the evaluation. Two metrics inspired from machine learning can be associated with a selected set *S* of decoys [27]: (1) *n* (number) and *p* (purity). The first measures the percentage of near-native decoys in *S* relative to the overall number of near-native decoys in the entire decoy dataset. The second measures the percentage of near-native decoys in *S* over the number of decoys in *S* itself. We note that *p* penalizes *S* if *S* contains a high number of false positives (non-native decoys) and so is a useful metric for decoy selection. If a set of decoys is presented to contain the true answer but the majority of decoys are false positives, then the ratio of signal to noise is too low to be useful for automated decoy selection. In contrast, a set with more near-native decoys is a better “prediction,” as the likelihood of selecting a near-native decoy uniformly at random from it is higher when the number of false positives is low.

We note that selecting a threshold dist_thresh RMSD allows populating the positive data set and carry out further evaluation. The threshold dist_thresh is set on a per-target basis, as there are protein targets on which the quality of generated decoys suffers from either the size and/or fold of the protein under investigation. We have taken care to consider proteins that are easy, medium, and hard in their difficulty for template-free structure prediction methods, such as Rosetta. For instance, we include in our evaluation cases where Rosetta does not get close to 3 Å of the known native structure. As we have done in previous work [27], we consider the following thresholds: If the lowest lRMSD min_dist (over all decoys) from a given native structure is ≤0.7 (these are considered easy cases), dist_thresh is set to 2 Å. Otherwise, dist_thresh is set to the minimum value that results in a non-zero number of near-native decoys in the largest-size cluster obtained via leader clustering; the latter is used as a baseline in our evaluation of community-based decoy selection. For medium-difficulty proteins (0.7 Å< min_dist <2 Å), dist_thresh varies between 2−4.5 Å. We set dist_thresh to 6 Å if min_dist ≥2 Å (these are the hard cases). This ensures a non-zero number of near-native decoys for evaluation. A detailed analysis of the impact of this threshold on cluster-based decoy selection is related in [27].

### 4.6. Implementation Details

Our in-house codes are implemented in Python. In the nngraph construction, δϵ = 0.2 Å, *k* = 20, $n_{\epsilon }$ = 5; $\epsilon_{0}$ is set so as not to exceed 900K edges, varying in 0.5−2.2 Å for most test cases, with one particularly challenging case for Rosetta set to 6.0 Å. The construction of the nngraph takes between 26 min to 4.25 h on one CPU. We consider different proximity-query data structures but select the kd-tree over the vp-tree for fast extraction of nearest neighbors, based on analysis of the time demands as a function of dimensionality (data not shown). The considered community detection methods take between 7 minutes and 35 hours using 8-cores and 1GB memory per core. We note that we consider 1≤c≤3 in community selection. 

## Figures and Tables

**Table 1:** Column 2 shows the PDB ID of a known native structure for each test case. Columns 3 and 4 show the fold (* indicates native structures with a predominant β fold and a short helix) and the length (number of amino acids), respectively. Column 5 shows the size of the decoy set Ω generated via Rosetta, and column 6 shows the lowest lRMSD from the known native structure over the decoy ensemble.

		PDB ID	Fold	Length (# aas)	|Ω|	min_dist (Å)
Easy	1.	1dtdb	α+β	61	57,839	0.51
	2.	1tig	α+β	88	52,099	0.60
	3.	1dtja	α+β	74	53,526	0.68
Medium	4.	1hz6a	α+β	64	57,474	0.72
	5.	1c8ca	$\beta^{*}$	64	53,322	1.08
	6.	1bq9	β	53	53,663	1.30
	7.	1sap	β	66	51,209	1.75
Hard	8.	2ezk	α	93	50,192	2.56
	9.	1aoy	α	78	52,218	3.26
	10.	1isua	coil	62	60,360	5.53

**Table 2:** The *s*, *n*, *p* of the sets of communities selected by different selection strategies over communities identified by the **Louvain** algorithm on decoy datasets embedded as directed ngraphs. We refer to this setting as **Louvain${}_{Directed}$**. Recall that *s* stands for size (number of decoys), and *n* and *p* are the two performance metrics described above (and in more detail in Section 4).

	Louvain${}_{\mathrm{Directed}}$			
	Sel-S	Sel-S+E	Sel-PR	Sel-PR+PC
	**s, n, p (%)**	**s, n, p (%)**	**s, n, p (%)**	**s, n, p (%)**
**1dtdb**	C${}_{1}$: 5.3, 23.4, 100	C${}_{1}$: 5.3, 23.4, 100	C${}_{1}$: 5.3, 23.4, 100	C${}_{1}$: 0.005, 0, 0
	C${}_{1-2}$: 10.3, 45.2, 100	C${}_{1-2}$: 5.5, 24.3, 100	C${}_{1-2}$: 5.3 24.3, 99.9	C${}_{1-2}$: 0.009, 0, 0
	C${}_{1-3}$: 15, 65.7, 100	C${}_{1-3}$: 10.2, 44.7, 100	C${}_{1-3}$: 5.4, 23.4, 99.7	C${}_{1-3}$: 5.3, 23.4, 99.8
**1tig**	C${}_{1}$: 10.4, 10.1, 14.6	C${}_{1}$: 10.4, 10.1, 14.6	C${}_{1}$: 10.4, 10.1, 14.6	C${}_{1}$: 10.4, 10.1, 14.6
	C${}_{1-2}$: 18.7, 18.4, 14.8	C${}_{1-2}$: 15.9, 15.6, 14.7	C${}_{1-2}$: 10.4, 10.1, 14.6	C${}_{1-2}$: 10.4, 10.1, 14.6
	C${}_{1-3}$: 24.2, 23.9, 14.9	C${}_{1-3}$: 17.7, 17.3, 14.8	C${}_{1-3}$: 10.4, 10.1, 14.6	C${}_{1-3}$: 10.4, 10.1, 14.6
**1dtja**	C${}_{1}$: 3.6, 16, 100	C${}_{1}$: 0.9, 3.9, 100	C${}_{1}$: 3.6, 16, 100	C${}_{1}$: 0.004, 0, 0
	C${}_{1-2}$: 6.6, 29.6, 100	C${}_{1-2}$: 3.6, 16, 100	C${}_{1-2}$: 6.3 28.1, 100	C${}_{1-2}$: 0.007, 0, 0
	C${}_{1-3}$: 9.4, 41.7, 100	C${}_{1-3}$: 5.2, 23, 100	C${}_{1-3}$: 7.2, 32, 100	C${}_{1-3}$: 0.01, 0.02, 33.3
**1hz6a**	C${}_{1}$: 6.4, 6.7, 11.7	C${}_{1}$: 3.9, 4.3, 12.6	C${}_{1}$: 6.4, 6.7, 11.7	C${}_{1}$: 0.02, 0.02, 9.1
	C${}_{1-2}$: 12, 12.4, 11.7	C${}_{1-2}$: 9.4, 8.9, 10.7	C${}_{1-2}$: 11.9, 11.2, 10.6	C${}_{1-2}$: 0.02, 0.02, 7.7
	C${}_{1-3}$: 17.5, 17, 11	C${}_{1-3}$: 13.9, 13.1, 10.6	C${}_{1-3}$: 15.8, 15.6, 11.1	C${}_{1-3}$: 0.03, 0.02, 6.7
**1c8ca**	C${}_{1}$: 4.6, 0.1, 0.1	C${}_{1}$: 2.9, 8.3, 31.7	C${}_{1}$: 4.6, 0.1, 0.1	C${}_{1}$: 0.006, 0, 0
	C${}_{1-2}$: 8.8, 6, 7.4	C${}_{1-2}$: 6, 32.8, 59.8	C${}_{1-2}$: 8.8, 6, 7.4	C${}_{1-2}$: 2.9, 8.3, 31.6
	C${}_{1-3}$: 12.5, 13.3, 11.6	C${}_{1-3}$: 7.5, 33.6, 48.8	C${}_{1-3}$: 11.9, 30.4, 27.8	C${}_{1-3}$: 6, 32.8, 59.7
**1sap**	C${}_{1}$: 11.4, 0, 0	C${}_{1}$: 8.5, 59.9, 16.3	C${}_{1}$: 11.4, 0, 0	C${}_{1}$: 8.5, 60, 16.3
	C${}_{1-2}$: 21.7, 0, 0	C${}_{1-2}$: 13.5, 62.5, 10.7	C${}_{1-2}$: 21.7, 0, 0	C${}_{1-2}$: 18.7, 60, 7.4
	C${}_{1-3}$: 30.6, 0.1, 0.01	C${}_{1-3}$: 23.8, 62.5, 6.1	C${}_{1-3}$: 30.2, 59.9, 4.6	C${}_{1-3}$: 18.7, 60, 7.4
**1bq9**	C${}_{1}$: 11.3, 9.9, 1.4	C${}_{1}$: 10.3, 11, 1.7	C${}_{1}$: 11.3, 9.9, 1.4	C${}_{1}$: 0.01, 0, 0
	C${}_{1-2}$: 21.6, 20.9, 1.5	C${}_{1-2}$: 21.6, 20.9, 1.5	C${}_{1-2}$: 21.6, 20.9, 1.5	C${}_{1-2}$: 0.02, 0, 0
	C${}_{1-3}$: 25.7, 25.2, 1.6	C${}_{1-3}$: 25.7, 25.5, 1.6	C${}_{1-3}$: 21.6, 20.9, 1.5	C${}_{1-3}$: 10.3, 11, 1.7
**2ezk**	C${}_{1}$: 30.1, 50.7, 22	C${}_{1}$: 30.1, 50.7, 22	C${}_{1}$: 30.1, 50.7, 22	C${}_{1}$: 30.1, 50.7, 22
	C${}_{1-2}$: 56.5, 56.2, 13	C${}_{1-2}$: 56.5, 56.2, 13	C${}_{1-2}$: 30.1, 50.7, 22	C${}_{1-2}$: 30.1, 50.7, 22
	C${}_{1-3}$: 73, 94.5, 16.9	C${}_{1-3}$: 60.8, 56.6, 12.1	C${}_{1-3}$: 30.1, 50.7, 22	C${}_{1-3}$: 30.1, 50.7, 22
**1aoy**	C${}_{1}$: 38, 22.6, 6.5	C${}_{1}$: 38, 22.6, 6.5	C${}_{1}$: 38, 22.6, 6.5	C${}_{1}$: 38, 22.6, 6.5
	C${}_{1-2}$: 54.9, 29.7, 5.9	C${}_{1-2}$: 52.5, 90.7, 18.9	C${}_{1-2}$: 38, 22.6, 6.5	C${}_{1-2}$: 38, 22.6, 6.5
	C${}_{1-3}$: 69.4, 97.9, 15.4	C${}_{1-3}$: 59.5, 92.7, 17	C${}_{1-3}$: 38, 22.6, 6.5	C${}_{1-3}$: 38, 22.6, 6.5
**1isua**	C${}_{1}$: 15.6, 57.3, 19.5	C${}_{1}$: 5, 0.7, 0.8	C${}_{1}$: 15.6, 57.3, 19.5	C${}_{1}$: 0.003, 0, 0
	C${}_{1-2}$: 26.3, 70.2, 14.2	C${}_{1-2}$: 9.9, 2, 1.1	C${}_{1-2}$: 22.5, 62.3, 14.7	C${}_{1-2}$: 0.007, 0, 0
	C${}_{1-3}$: 33.2, 75.2, 12	C${}_{1-3}$: 15.9, 3.6, 1.2	C${}_{1-3}$: 28.5, 63.9, 11.9	C${}_{1-3}$: 0.01, 0, 0

**Table 3:** The *s*, *n*, *p* of the communities selected by different selection strategies over communities identified by the **Louvain** algorithm on decoy datasets embedded as undirected ngraphs.

	Louvain			
	Sel-S	Sel-S+E	Sel-PR	Sel-PR+PC
	s, n, p (%)	s, n, p (%)	s, n, p (%)	s, n, p (%)
**1dtdb**	C${}_{1}$: 4.7, 20.4, 100	C${}_{1}$: 2.5, 10.8, 100	C${}_{1}$: 3.1, 13.7, 100	C${}_{1}$: 0.01, 0, 0
	C${}_{1-2}$: 8.2, 35.9, 99.9	C${}_{1-2}$: 5.1, 22.2, 100	C${}_{1-2}$: 7.8, 34.1, 100	C${}_{1-2}$: 0.01, 0, 0
	C${}_{1-3}$: 11.3, 49.5, 99.9	C${}_{1-3}$: 7.2, 31.5, 100	C${}_{1-3}$: 10.2, 44.9, 100	C${}_{1-3}$: 2.5, 10.8, 99.6
**1tig**	C${}_{1}$: 12.6, 12.3, 14.7	C${}_{1}$: 12.6, 12.3, 14.7	C${}_{1}$: 12.6, 12.3, 14.7	C${}_{1}$: 12.6, 12.3, 14.7
	C${}_{1-2}$: 23, 23, 15	C${}_{1-2}$: 16.4, 16, 14.7	C${}_{1-2}$: 12.6, 12.3, 14.7	C${}_{1-2}$: 12.6, 12.4, 14.8
	C${}_{1-3}$: 28, 28.1, 15.1	C${}_{1-3}$: 18, 17.7, 14.8	C${}_{1-3}$: 12.6, 12.3, 14.7	C${}_{1-3}$: 12.6, 12.4, 14.8
**1dtja**	C${}_{1}$: 3.2, 14.2, 100	C${}_{1}$: 1.4, 6.2, 100	C${}_{1}$: 3.2, 14.2, 100	C${}_{1}$: 0.004, 0, 0
	C${}_{1-2}$: 6.3, 28, 100	C${}_{1-2}$: 3.1, 13.6, 100	C${}_{1-2}$: 6.3, 28, 100	C${}_{1-2}$: 0.007, 0, 0
	C${}_{1-3}$: 9, 40.1, 100	C${}_{1-3}$: 5.8, 25.7, 100	C${}_{1-3}$: 9, 40.1, 100	C${}_{1-3}$: 0.01, 0.02, 33.3
**1hz6a**	C${}_{1}$: 7, 7.1, 11.4	C${}_{1}$: 5.8, 5.9, 11.5	C${}_{1}$: 6, 6, 11.3	C${}_{1}$: 0.02, 0.02, 9.1
	C${}_{1-2}$: 13, 13.1, 11.3	C${}_{1-2}$: 11.1, 10.7, 10.9	C${}_{1-2}$: 11.8, 11.9, 11.4	C${}_{1-2}$: 0.02, 0.02, 7.7
	C${}_{1-3}$: 18.8, 19, 11.4	C${}_{1-3}$: 17.1, 16.6, 11	C${}_{1-3}$: 18.8, 19, 11.4	C${}_{1-3}$: 0.03, 0.02, 6.7
**1c8ca**	C${}_{1}$: 4.7, 0.1, 0.2	C${}_{1}$: 2.7, 8.5, 34.8	C${}_{1}$: 2.7, 8.5, 34.8	C${}_{1}$: 0.006, 0, 0
	C${}_{1-2}$: 8.6, 32.4, 41.1	C${}_{1-2}$: 6.6, 40.8, 67.5	C${}_{1-2}$: 7.3, 8.6, 12.8	C${}_{1-2}$: 2.7, 8.5, 34.7
	C${}_{1-3}$: 12.2, 42.8, 38.2	C${}_{1-3}$: 9.9, 47.3, 51.8	C${}_{1-3}$: 11.2, 40.9, 39.6	C${}_{1-3}$: 6.6, 40.8, 67.4
**1sap**	C${}_{1}$: 10.1, 0, 0	C${}_{1}$: 8.8, 59.6, 15.6	C${}_{1}$: 9.9, 0, 0	C${}_{1}$: 8.8, 59.6, 15.6
	C${}_{1-2}$: 20, 0, 0	C${}_{1-2}$: 14.2, 64.1, 10.4	C${}_{1-2}$: 20, 0, 0	C${}_{1-2}$: 18.7, 59.6, 7.3
	C${}_{1-3}$: 28.8, 59.6, 4.8	C${}_{1-3}$: 24.1, 64.1, 6.1	C${}_{1-3}$: 28.8, 59.6, 4.8	C${}_{1-3}$: 28.8, 59.6, 4.8
**1bq9**	C${}_{1}$: 11.3, 9.7, 1.4	C${}_{1}$: 10.3, 11.2, 1.7	C${}_{1}$: 10.3, 11.2, 1.7	C${}_{1}$: 0.01, 0, 0
	C${}_{1-2}$: 21.7, 20.9, 1.5	C${}_{1-2}$: 21.7, 20.9, 1.5	C${}_{1-2}$: 21.7, 20.9, 1.5	C${}_{1-2}$: 0.02, 0, 0
	C${}_{1-3}$: 26.2, 25.6, 1.5	C${}_{1-3}$: 26.2, 25.7, 1.6	C${}_{1-3}$: 21.7, 20.9, 1.5	C${}_{1-3}$: 10.4, 11.2, 1.7
**2ezk**	C${}_{1}$: 26.9, 47.2, 22.9	C${}_{1}$: 26.9, 47.2, 22.9	C${}_{1}$: 26.9, 47.2, 22.9	C${}_{1}$: 26.9, 47.2, 22.9
	C${}_{1-2}$: 53.1, 52.3, 12.8	C${}_{1-2}$: 53.2, 52.3, 12.8	C${}_{1-2}$: 26.9, 47.2, 22.9	C${}_{1-2}$: 26.9, 47.2, 22.9
	C${}_{1-3}$: 69.2, 81.8, 15.4	C${}_{1-3}$: 58.1, 52.7, 11.8	C${}_{1-3}$: 26.9, 47.2, 22.9	C${}_{1-3}$: 26.9, 47.2, 22.9
**1aoy**	C${}_{1}$: 36.8, 27.6, 8.2	C${}_{1}$: 36.8, 27.6, 8.2	C${}_{1}$: 36.8, 27.6, 8.2	C${}_{1}$: 36.8, 27.6, 8.2
	C${}_{1-2}$: 53.1, 33.7, 6.9	C${}_{1-2}$: 52.4, 91.6, 19.1	C${}_{1-2}$: 36.8, 27.6, 8.2	C${}_{1-2}$: 36.8, 27.6, 8.2
	C${}_{1-3}$: 68.8, 97.7, 15.5	C${}_{1-3}$: 65, 91.9, 15.5	C${}_{1-3}$: 36.8, 27.6, 8.2	C${}_{1-3}$: 36.8, 27.6, 8.2
**1isua**	C${}_{1}$: 14.3, 56, 20.7	C${}_{1}$: 5.5, 1.3, 1.2	C${}_{1}$: 5.6, 1.5, 1.4	C${}_{1}$: 0.003, 0, 0
	C${}_{1-2}$: 23.9, 64.5, 14.3	C${}_{1-2}$: 11.2, 2.8, 1.3	C${}_{1-2}$: 13.5, 6.4, 2.5	C${}_{1-2}$: 0.007, 0, 0
	C${}_{1-3}$: 31.8, 69.3, 11.6	C${}_{1-3}$: 16.3, 6, 2	C${}_{1-3}$: 27.8, 62.4, 11.9	C${}_{1-3}$: 0.01, 0, 0

**Table 4:** The *s*, *n*, *p* of the communities selected by different strategies over communities identified by the Greedy Modularity Maximization (GMM) algorithm on decoy datasets embedded as undirected graphs.

	GMM			
	Sel-S	Sel-S+E	Sel-PR	Sel-PR+PC
	s, n, p (%)	s, n, p (%)	s, n, p (%)	s, n, p (%)
**1dtdb**	C${}_{1}$: 11.1, 48.7, 100	C${}_{1}$: 11.1, 48.7, 100	C${}_{1}$: 11.1, 48.7, 100	C${}_{1}$: 0.005, 0, 0
	C${}_{1-2}$: 18.3, 80.1, 99.9	C${}_{1-2}$: 14.8, 65, 100	C${}_{1-2}$: 11.1, 48.7, 99.9	C${}_{1-2}$: 0.009, 0, 0
	C${}_{1-3}$: 22, 96.4, 99.9	C${}_{1-3}$: 15.3, 65, 96.8	C${}_{1-3}$: 11.1, 48.7, 99.9	C${}_{1-3}$: 0.02, 0, 0
**1tig**	C${}_{1}$: 19.2, 18.7, 14.7	C${}_{1}$: 19.2, 18.7, 14.7	C${}_{1}$: 19.2, 18.7, 14.7	C${}_{1}$: 19.2, 18.7, 14.7
	C${}_{1-2}$: 31.6, 31.5, 15	C${}_{1-2}$: 20.4, 20, 14.8	C${}_{1-2}$: 19.2, 18.7, 14.7	C${}_{1-2}$: 19.2, 18.7, 14.7
	C${}_{1-3}$: 43, 42.3, 14.8	C${}_{1-3}$: 22.9, 22.3, 14.7	C${}_{1-3}$: 19.2, 18.7, 14.7	C${}_{1-3}$: 19.2, 18.7, 14.7
**1dtja**	C${}_{1}$: 7.6, 33.7, 100	C${}_{1}$: 0.03, 0.1, 100	C${}_{1}$: 7.6, 33.7, 100	C${}_{1}$: 0.004, 0, 0
	C${}_{1-2}$: 14.5, 64.6, 100	C${}_{1-2}$: 7.6, 33.9, 100	C${}_{1-2}$: 7.6, 33.9, 100	C${}_{1-2}$: 0.007, 0, 0
	C${}_{1-3}$: 17.6, 78.5, 100	C${}_{1-3}$: 14.5, 64.7, 100	C${}_{1-3}$: 7.6, 33.9, 100	C${}_{1-3}$: 0.01, 0.02, 33.3
**1hz6a**	C${}_{1}$: 24.7, 24.8, 11.3	C${}_{1}$: 24.7, 24.8, 11.3	C${}_{1}$: 24.7, 24.8, 11.3	C${}_{1}$: 0.02, 0.02, 9.1
	C${}_{1-2}$: 48.7, 48.8, 11.3	C${}_{1-2}$: 25.7, 25.7, 11.3	C${}_{1-2}$: 24.7, 24.8, 11.3	C${}_{1-2}$: 0.03, 0.03, 12.5
	C${}_{1-3}$: 59, 59.2, 11.3	C${}_{1-3}$: 28.3, 28.4, 11.3	C${}_{1-3}$: 24.7, 24.8, 11.3	C${}_{1-3}$: 0.03, 0.05, 15.8
**1c8ca**	C${}_{1}$: 11.6, 6.9, 6.5	C${}_{1}$: 6.4, 15.7, 26.5	C${}_{1}$: 11.6, 6.9, 6.5	C${}_{1}$: 0.006, 0, 0
	C${}_{1-2}$: 23.1, 60.9, 28.7	C${}_{1-2}$: 17.9, 69.7, 42.3	C${}_{1-2}$: 23.1, 60.9, 28.7	C${}_{1-2}$: 0.06, 0.5, 91.2
	C${}_{1-3}$: 29.5, 76.6, 28.3	C${}_{1-3}$: 18.3, 69.7, 41.4	C${}_{1-3}$: 29.5, 76.6, 28.3	C${}_{1-3}$: 6.5, 16.2, 27.2
**1sap**	C${}_{1}$: 26, 0, 0	C${}_{1}$: 24.6, 99.3, 9.3	C${}_{1}$: 26, 0, 0	C${}_{1}$: 0.01, 0, 0
	C${}_{1-2}$: 50.6, 99.3, 4.5	C${}_{1-2}$: 40.5, 99.3, 5.7	C${}_{1-2}$: 50.6, 99.3, 4.5	C${}_{1-2}$: 24.6, 99.3, 9.3
	C${}_{1-3}$: 66.5, 99.3, 3.4	C${}_{1-3}$: 66.6, 99.3, 3.4	C${}_{1-3}$: 50.6, 99.3, 4.5	C${}_{1-3}$: 24.6, 99.3, 9.3
**1bq9**	C${}_{1}$: 24.5, 23.8, 1.5	C${}_{1}$: 24.5, 23.8, 1.5	C${}_{1}$: 24.5, 23.8, 1.5	C${}_{1}$: 0.01, 0, 0
	C${}_{1-2}$: 32.6, 32.4, 1.6	C${}_{1-2}$: 24.9, 24.1, 1.5	C${}_{1-2}$: 24.5, 23.9, 1.6	C${}_{1-2}$: 0.02, 0, 0
	C${}_{1-3}$: 38.7, 38.7, 1.6	C${}_{1-3}$: 26.9, 26.1, 1.5	C${}_{1-3}$: 24.5, 23.9, 1.6	C${}_{1-3}$: 0.03, 0.1, 7.1
**2ezk**	C${}_{1}$: 43.1, 7, 2.1	C${}_{1}$: 22.4, 40, 23.3	C${}_{1}$: 43.1, 7, 2.1	C${}_{1}$: 22.4, 40, 23.3
	C${}_{1-2}$: 76.6, 60, 10.2	C${}_{1-2}$: 65.5, 47, 9.4	C${}_{1-2}$: 65.5, 47, 9.4	C${}_{1-2}$: 65.5, 47, 9.4
	C${}_{1-3}$: 99, 100, 13.2	C${}_{1-3}$: 65.5, 47, 9.4	C${}_{1-3}$: 65.5, 47, 9.4	C${}_{1-3}$: 65.5, 47, 9.4
**1aoy**	C${}_{1}$: 45.6, 66.8, 16	C${}_{1}$: 35.6, 33, 10.1	C${}_{1}$: 45.6, 66.8, 16	C${}_{1}$: 35.6, 33, 10.1
	C${}_{1-2}$: 81.2, 99.7, 13.1	C${}_{1-2}$: 81.2, 99.7, 13.4	C${}_{1-2}$: 81.2, 99.7, 13.4	C${}_{1-2}$: 35.6, 33, 10.1
	C${}_{1-3}$: 96.8, 100, 11.3	C${}_{1-3}$: 81.2, 99.7, 13.4	C${}_{1-3}$: 81.2, 99.7, 13.4	C${}_{1-3}$: 81.2, 99.7, 13.4
**1isua**	C${}_{1}$: 39.6, 70, 9.4	C${}_{1}$: 14.1, 1.9, 0.7	C${}_{1}$: 39.6, 70, 9.4	C${}_{1}$: 0.007, 0, 0
	C${}_{1-2}$: 78.7, 97.7, 6.6	C${}_{1-2}$: 14.8, 1.9, 0.7	C${}_{1-2}$: 53.7, 71.9, 7.1	C${}_{1-2}$: 0.01, 0, 0
	C${}_{1-3}$: 92.9, 99.5, 5.7	C${}_{1-3}$: 54.4, 71.9, 7	C${}_{1-3}$: 53.8, 71.9, 7.1	C${}_{1-3}$: 0.02, 0, 0

**Table 5:** Rank (by Size(S), Size and Energy(S+E), Pareto rank(PR), Pareto rank and Pareto count (PR+PC)) of the community with the highest purity among those identified by Louvain (Lo), Louvain${}_{Directed}$ (Lo${}_{D}$) and GMM.

	Rank by (Lo)	Rank by (Lo${}_{D}$)	Rank by (GMM)
	S, S+E, PR, PR+PC	S, S+E, PR, PR+PC	S, S+E, PR, PR+PC
**1dtdb**	3, 4, **1**, 9	**1**, **1**, **1**, 3	**1**, **1**, **1**, 8
**1tig**	691, **396**, 7069, 7073	229, **112**, 2287, 2289	283, **44**, 962, 963
**1dtja**	71, **64**, 26735, 26736	**1**, 7, **1**, 12	**1**, 3, **1**, 9
**1hz6a**	647, **639**, 10160, 10166	280, **49**, 673, 670	337, **70**, 748, 740
**1c8ca**	818, **572**, 9700, 9736	42, **31**, 540, 542	15, **1**, 4, 2
**1bq9**	1230, **267**, 4816, 4836	1223, **268**, 4810, 4827	1271, **269**, 4826, 4853
**1sap**	3301, **137**, 538, 541	3298, **137**, 538, 551	3369, **142**, 566, 566
**2ezk**	6, **5**, 13, 12	**3**, 9, 14, 16	3, **1**, 2, **1**
**1aoy**	3, **2**, 12, 11	**3**, **3**, 14, 13	**1**, 3, **1**, 3
**1isua**	135, **117**, 1519, 1527	136, **117**, 1520, 1525	194, **193**, 1236, 1241

**Table 6:** Comparison of Size + Energy (S+E) to other selection strategies on best rank via 1-sided Fisher’s and Barnard’s tests. Top panel evaluates the null hypothesis that Sel-S+E does not provide the best rank (based on reported p-values), considering each of the other three selection strategies in turn. Similarly, the lower panel evaluates the null hypothesis that Sel-S+E does not provide a better rank with respect to another particular selection strategy, considering each in turn.

**Best Rank**			
**Test**	**Sel–S**	**Sel–PR**	**Sel–PR+PC**
Fisher’s	6.621 × 10${}^{-7}$	1.626 × 10${}^{-7}$	9.388 × 10${}^{-12}$
Barnard’s	2.314 × 10${}^{-7}$	6.33 × 10${}^{-8}$	2.128 × 10${}^{-12}$
**Better Rank**			
**Test**	**Sel–S**	**Sel–PR**	**Sel–PR+PC**
Fisher’s	0.0001154	7.744 × 10${}^{-7}$	4.194 × 10${}^{-15}$
Barnard’s	6.738 × 10${}^{-5}$	3.811 × 10${}^{-7}$	8.075 × 10${}^{-16}$

**Table 7:** Comparison of **Size + Energy** to other selection strategies on best rank via **2-sided** Fisher’s and Barnard’s tests. The tests evaluate the null hypothesis (based on reported p-values) that **Sel-S+E** (or, **Size+Energy**) provides similar ranking in comparison to other selection strategies.

**Best Rank**			
**Test**	**Sel–S**	**Sel–PR**	**Sel–PR+PC**
Fisher’s	1.324 × 10${}^{-6}$	3.252 × 10${}^{-7}$	1.878 × 10${}^{-11}$
Barnard’s	4.629 × 10${}^{-7}$	1.266 × 10${}^{-7}$	4.255 × 10${}^{-12}$
**Better Rank**			
**Test**	**Sel–S**	**Sel–PR**	**Sel–PR+PC**
Fisher’s	0.000231	1.549 × 10${}^{-6}$	8.388 × 10${}^{-15}$
Barnard’s	0.0001348	7.621 × 10${}^{-7}$	1.615 × 10${}^{-15}$

**Table 8:** Entropy values for the Louvain and GMM methods.

	Entropy${}_{\mathrm{Lo}(\mathrm{Undirected})}$	Entropy${}_{\mathrm{GMM}(\mathrm{Undirected})}$	Entropy${}_{\mathrm{Lo}(\mathrm{Directed})}$
**1dtdb**	2.054332	**1.335355**	2.007146
**1tig**	5.571811	**5.19006**	5.660448
**1dtja**	3.679291	**2.990441**	3.670991
**1hz6a**	4.2847	**3.472866**	4.400298
**1c8ca**	3.323009	**2.713008**	3.480973
**1bq9**	4.920814	**4.575263**	4.92005
**1sap**	0.961949	**0.054711**	0.914548
**2ezk**	1.222933	**0.888501**	1.065169
**1aoy**	0.905185	**0.652051**	0.873628
**1isua**	1.725124	**0.715579**	1.680263

## Supplementary Materials

The following are available online. Supplementary Figure S1: Comparison of community detection methods (encoded by different colors) on undirected nngraphs embedding each of the 10 decoy datasets along (a) Modularity, (b) Conductance, (c) Max odf (out degree fraction); Figure S2: Comparison of community detection methods (encoded by different colors) on undirected nngraphs embedding each of the 10 decoy datasets along (a) expansion, (b) cut ratio, (c) average odf (out degree fraction); Figure S3: Comparison of community detection methods (encoded by different colors) on directed nngraphs embedding each of the 10 decoy datasets along (a) expansion, (b) cut ratio, (c) average odf (out degree fraction); Figure S4: Comparison of the various selection strategies on the percentage of near-natives of the top 3 communities C1−3 selected over communities detected with the Louvain method on directed nngraph embeddings of decoy data in (a), the Louvain method on undirected nngraph embeddings of decoy data in (b), and the GMM method on undirected nngraph embeddings of decoy data in (c); Figure S5: Comparison of the various selection strategies on the purity of the top 3 communities C1−3 selected over communities detected with the Louvain method on directed nngraph embeddings of decoy data in (a), the Louvain method on undirected nngraph embeddings of decoy data in (b), and the GMM method on undirected nngraph embeddings of decoy data in (c).

## Abbreviations

The following abbreviations are used in this manuscript:

GMM	Greedy Modularity Maximization
IM	InfoMap
LE	Leading Eigenvector
Lo	Louvain
LP	Label Propagation
PDB	Protein Data Bank
CASP	Critical Assessment of protein Structure Prediction
lRMSD	least root-mean-squared-deviation
ML	Machine Learning
PC	Pareto Count
PR	Pareto Rank
SVM	Support Vector Machines
WT	Walktrap
