# Supplementary material for: Graph-Based Community Detection for Decoy Selection in Template-Free Protein Structure Prediction
Source: Molecules. 2019 Feb 28;24(5):854. doi: 10.3390/molecules24050854 (PMC6429114; doi:10.3390/molecules24050854)
Supplement: Supplementary file 1 [file molecules-24-00854-s001.pdf]

Supplementary Material: Graph-based Community Detection for Decoy Selection in Template-free Protein Structure Prediction

Kazi Lutful Kabir, Liban Hassan, Zahra Rajabi,
Nasrin Akhter, and Amarda Shehu

Metrics Evaluating Community Detection Methods

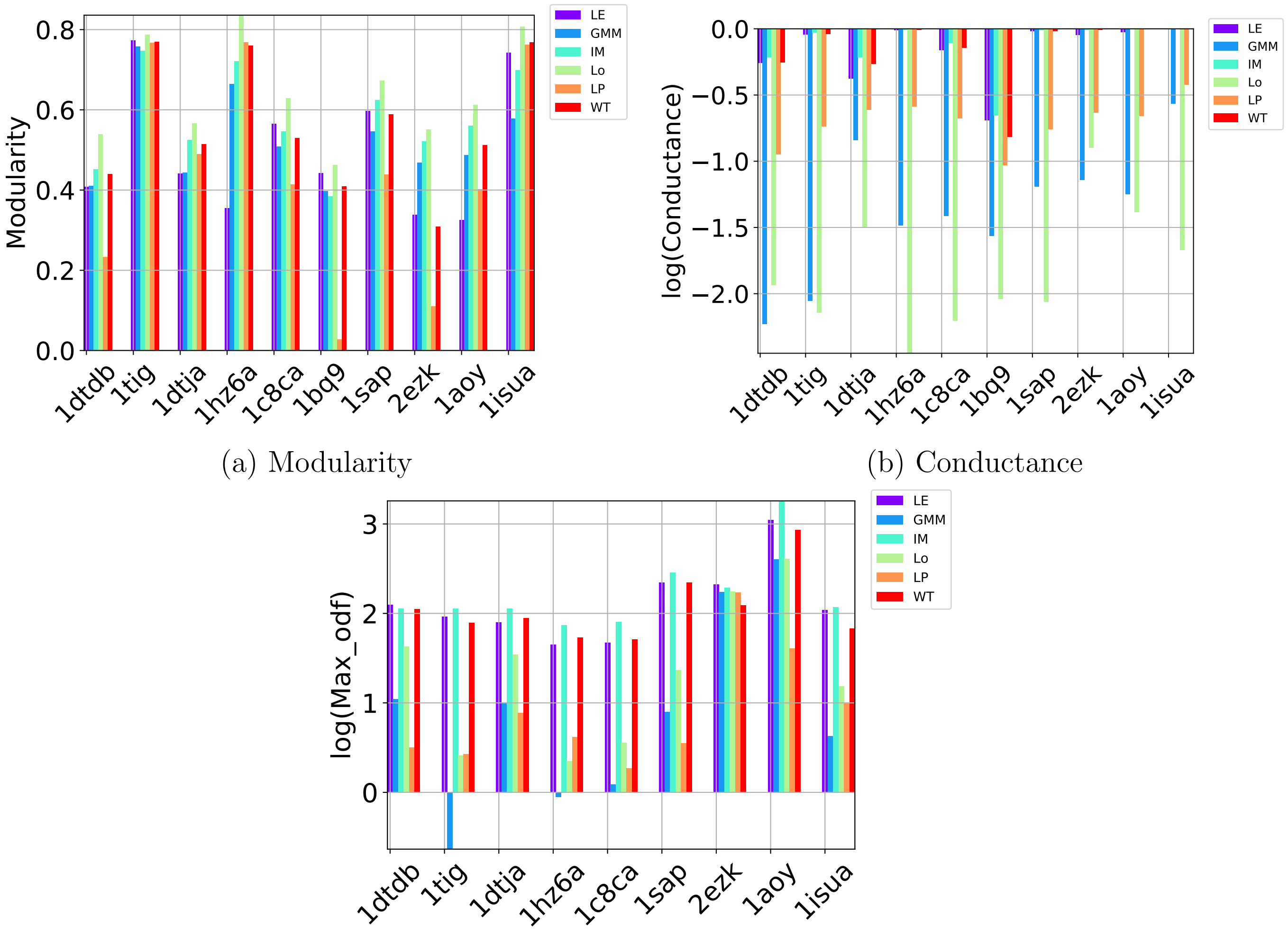

(a) Modularity

(b) Conductance

(c) Max odf

Figure 1: Comparison of community detection methods (encoded by different colors) on undirected nngraphs embedding each of the 10 decoy datasets along (a) Modularity, (b) Conductance, (c) Max odf (out degree fraction).

Figures 1 and 2 show the comparison along additional community evaluation metrics that evaluate communities over undirected nngraphs. The shown results support the observations made in the main article that Louvain and GMM yield better communities. We recall that better communities relate with higher modularity, lower conductance, and lower max odf.

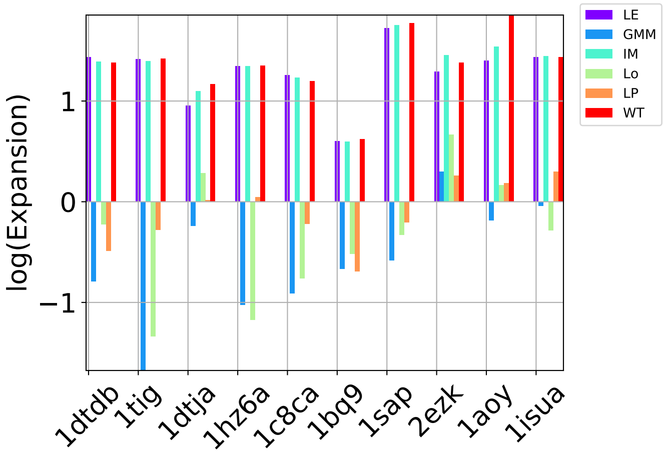

(a) Expansion

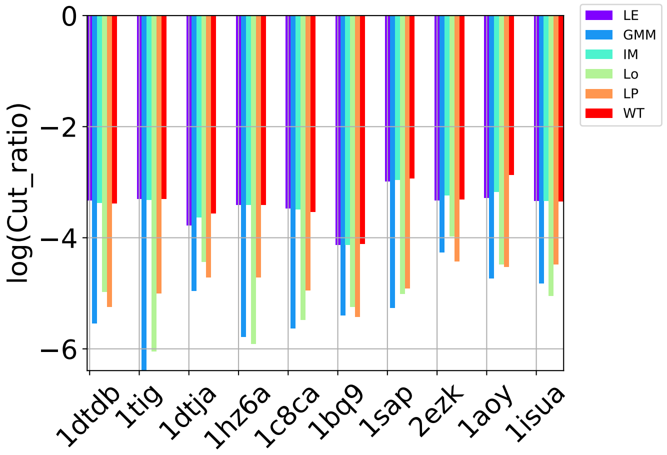

(b) Cut ratio

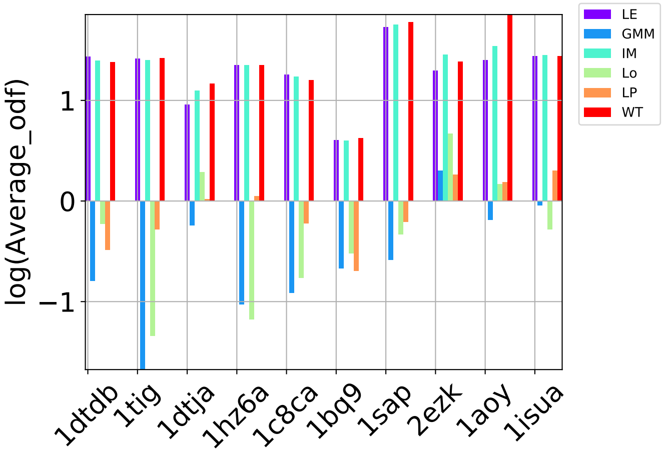

(c) Average odf

Figure 2: Comparison of community detection methods (encoded by different colors) on undirected nngraphs embedding each of the 10 decoy datasets along (a) expansion, (b) cut ratio, (c) average odf (out degree fraction).

Figure 3 compares the Louvain, Walktrap and InfoMap community detection techniques on directed nngraphs along expansion, cut ratio, and average odf (out degree fraction). All these methods perform comparably on these metrics.

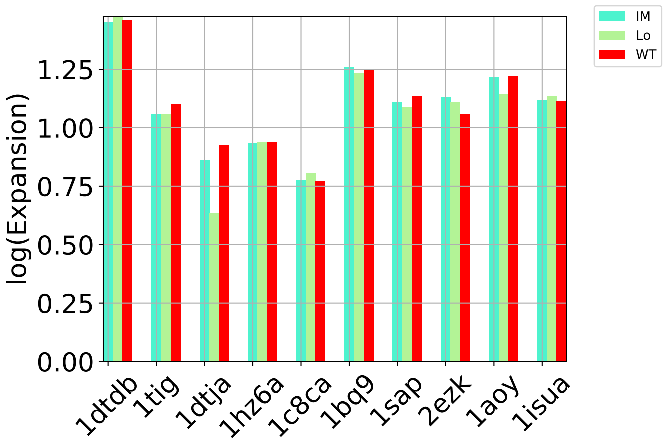

(a) Expansion

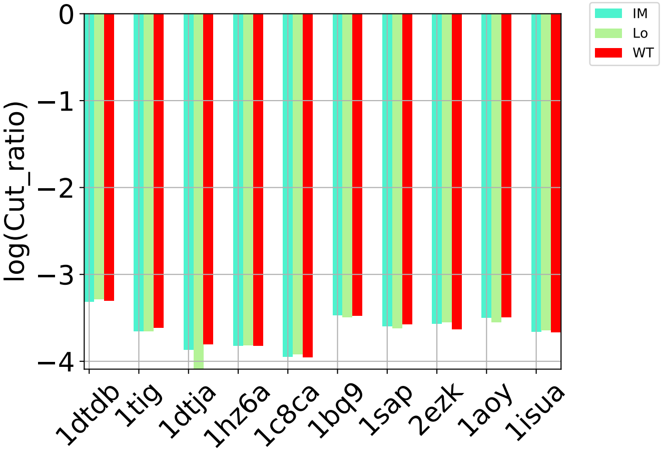

(b) Cut ratio

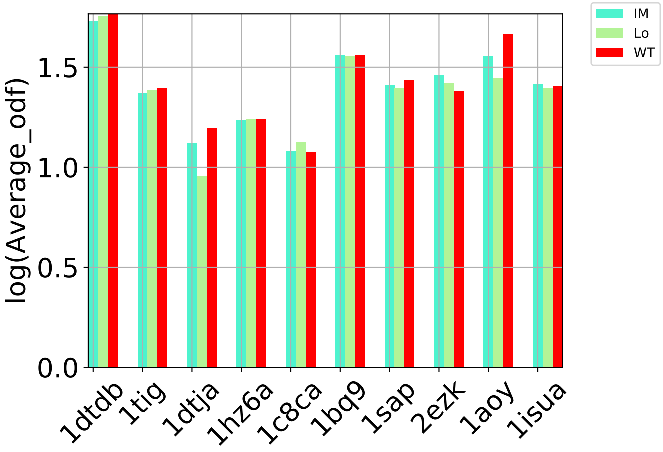

(c) Average odf

Figure 3: Comparison of community detection methods (encoded by different colors) on directed nngraphs embedding each of the 10 decoy datasets along (a) expansion, (b) cut ratio, (c) average odf (out degree fraction).

Evaluation of Community Selection Strategies for Decoy Selection

Figure 4 compares the selection strategies in terms of the percentage of near-native structures in the top 3 communities detected with the Louvain method on directed nngraph embeddings of decoy data in (a), the Louvain method on undirected nngraph embeddings of decoy data in (b), and the GMM method on undirected nngraph embeddings of decoy data in (c). The figures bolster the fact that Sel-S and Sel-S+E are better strategies with respect to others. Figure 5 shows the comparison of the selection strategies in terms of the purity of the top 3 communities detected with the Louvain method on directed nngraph embeddings of decoy data in (a), the Louvain method on undirected nngraph embeddings of decoy data in (b), and the GMM method on undirected nngraph embeddings of decoy data in (c). This comparison imply that, of the four selection strategies, Sel-S and Sel-S+E consistently yield good results. And taking all together, Sel-S+E is better than the others.

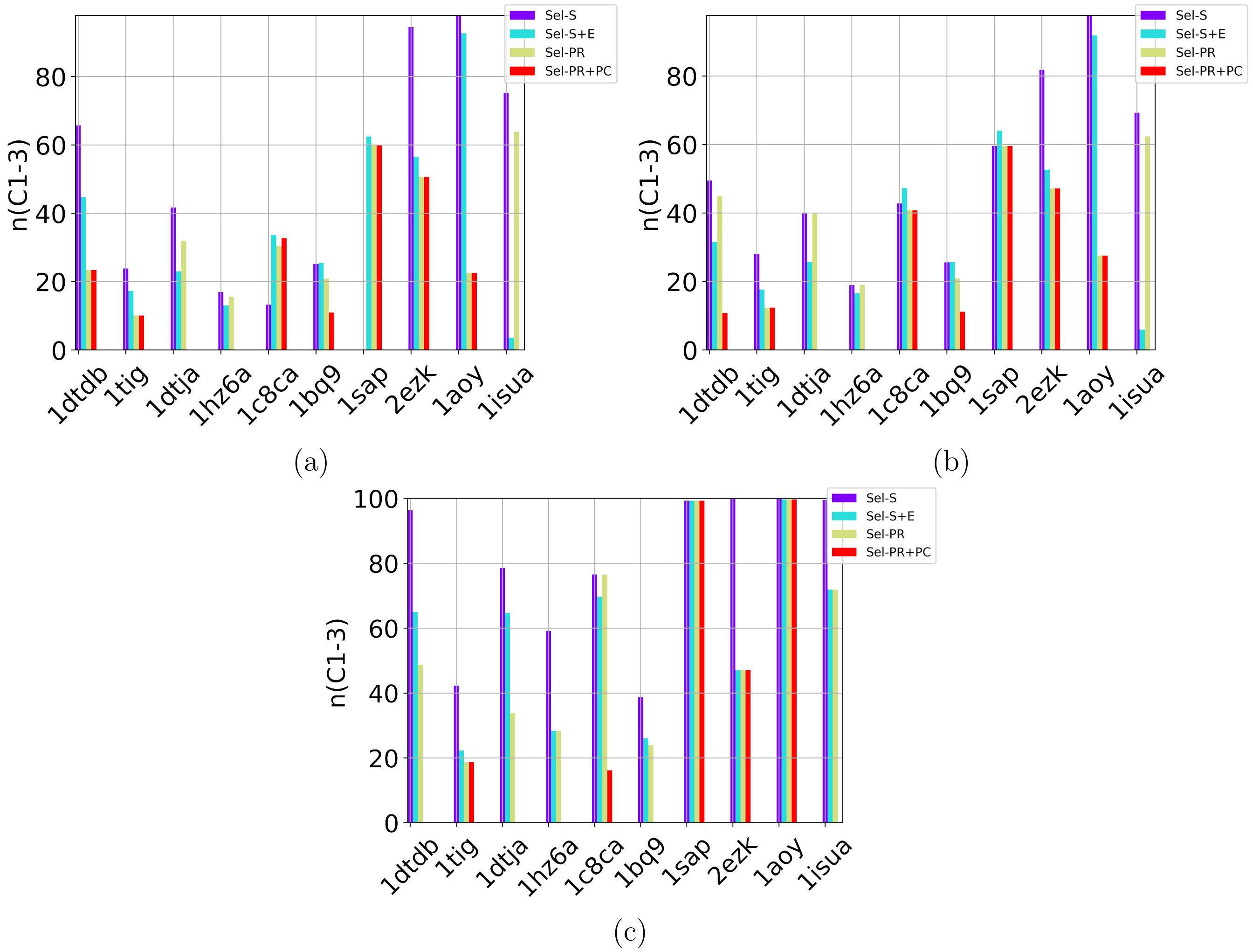

Figure 4: Comparison of the various selection strategies on the percentage of near-natives of the top 3 communities $C1-3$ selected over communities detected with the Louvain method on directed nngraph embeddings of decoy data in (a), the Louvain method on undirected nngraph embeddings of decoy data in (b), and the GMM method on undirected nngraph embeddings of decoy data in (c).

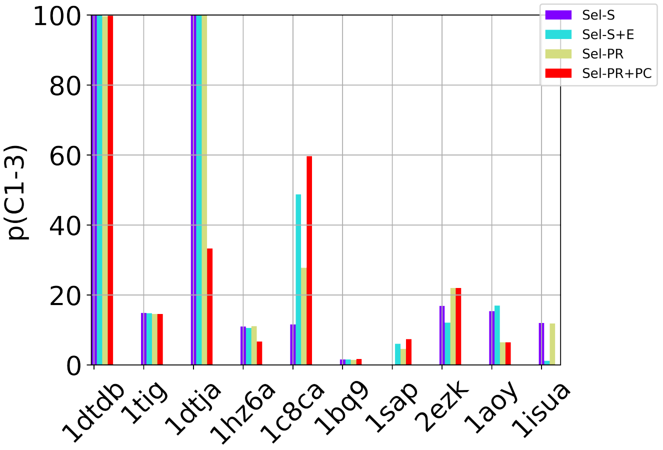

(a)

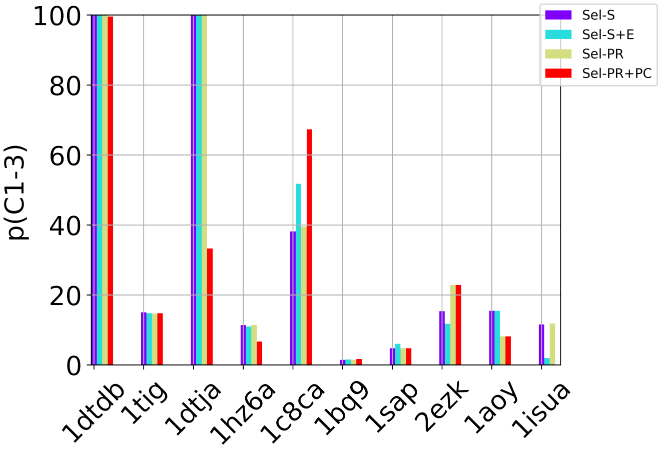

(b)

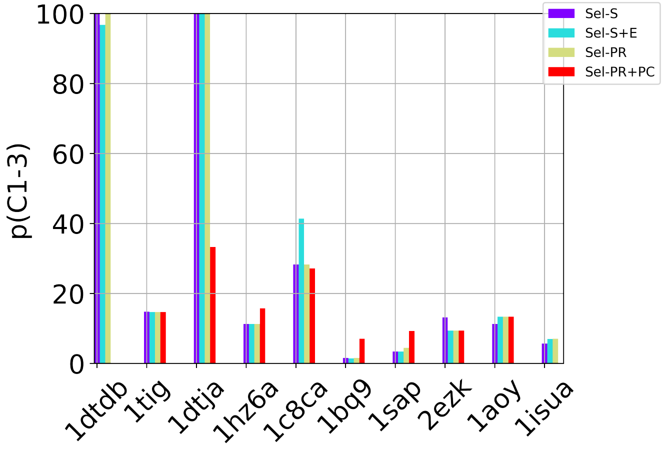

(c)

Figure 5: Comparison of the various selection strategies on the purity of the top 3 communities $C1-3$ selected over communities detected with the Louvain method on directed nngraph embeddings of decoy data in (a), the Louvain method on undirected nngraph embeddings of decoy data in (b), and the GMM method on undirected nngraph embeddings of decoy data in (c).
